# Fully-Actuated Aerial Manipulator for Infrastructure Contact Inspection: Design, Modeling, Localization, and Control

**DOI:** 10.3390/s20174708

**Published:** 2020-08-20

**Authors:** Pedro J. Sanchez-Cuevas, Antonio Gonzalez-Morgado, Nicolas Cortes, Diego B. Gayango, Antonio E. Jimenez-Cano, Aníbal Ollero, Guillermo Heredia

**Affiliations:** 1 Robotics, Vision and Control Group, Universidad de Sevilla, Camino de los Descubrimientos s.n., 41092 Sevilla, Spain; antgonmor5@alum.us.es (A.G.-M.); niccorfer@alum.us.es (N.C.); diebengay@alum.us.es (D.B.G.); aollero@us.es (A.O.); guiller@us.es (G.H.); 2 Laboratory for Analysis and Architecture of Systems, Centre National de la Recherche Scientifique, 7 Avenue du Colonel Roche, 31400 Toulouse, France; aejimenezc@laas.fr

**Keywords:** aerial systems, applications, inspection robotics, bridge inspection with UAS

## Abstract

This paper presents the design, modeling and control of a fully actuated aerial robot for infrastructure contact inspection as well as its localization system. Health assessment of transport infrastructure involves measurements with sensors in contact with the bridge and tunnel surfaces and the installation of monitoring sensing devices at specific points. The design of the aerial robot presented in the paper includes a 3DoF lightweight arm with a sensorized passive joint which can measure the contact force to regulate the force applied with the sensor on the structure. The aerial platform has been designed with tilted propellers to be fully actuated, achieving independent attitude and position control. It also mounts a “docking gear” to establish full contact with the infrastructure during the inspection, minimizing the measurement errors derived from the motion of the aerial platform and allowing full contact with the surface regardless of its condition (smooth, rough, ...). The localization system of the aerial robot uses multi-sensor fusion of the measurements of a topographic laser sensor on the ground and a tracking camera and inertial sensors on-board the aerial robot, to be able to fly under the bridge deck or close to the bridge pillars where GNSS satellite signals are not available. The paper also presents the modeling and control of the aerial robot. Validation experiments of the localization system and the control system, and with the aerial robot inspecting a real bridge are also included.

## 1. Introduction

In the last few years, the interest in Unmanned Aerial Vehicles (UAVs) has been continuously growing [1]. Especially during the last decade, the robotic community has paid attention to modeling, design, and control of multirotors [2], and then, due to their mechanical simplicity and availability of actuators, sensors, and electronics, their use has been multiplying in many areas with a huge variety of applications. The first cases of the use of these aerial robots were focused on perceiving, monitoring, or filming the environment. However, in recent years, the appearance of robotic aerial manipulation has widened the application range of these UAVs. Aerial manipulators [3,4] are aerial robots with arms or mechanical devices which are able to physically interact with the environment, performing tasks as object manipulation [5,6], operating devices [7,8], surface repairing [9], and taking measurements with sensors in contact with surfaces [10].

One of these applications in which the use of UAVs is being increasingly sought is the maintenance and safe operation of the existing civil infrastructure like bridges, viaducts, and tunnels, whose progressive deterioration requires in many cases inspection, assessment, and repair work.

Infrastructure inspection is done in most cases with non-destructive testing (NDT) methods. Visual inspection is one of the main methods used for inspection of the concrete walls of bridges and tunnels, which allow for detecting and identifying defects and the overall state of the infrastructure. On the other hand, sonic and ultrasonic sensors are used to measure crack depth and width and characterize the condition of the infrastructure concrete elements. Ultrasonic methods need the sensor be directly in contact with the surface of the bridge or tunnel and applying a constant force during the measurements.

Over the last decade, UAVs have been used for this purpose, and now there are prototypes [11,12] and commercially available drones for an initial, non-contact inspection of difficult to access areas of the infrastructure, mostly using imagery, videos [13,14,15,16], or three-dimensional point clouds [17]. In all these cases, though, the inspection is carried out remotely, and when a damage of concern is being detected, an in-depth inspection has to follow with experienced inspectors in need of hands-on-access to the infrastructure section.

Infrastructure inspection and maintenance has been one of the priority robotics research areas of the Horizon 2020 framework program. In particular, the H2020 AEROBI project has addressed successfully the problem of performing concrete bridge contact inspection with aerial robots and aerial manipulators [18]. The ongoing RESIST project is extending their use also for tunnel inspection and steel bridges [19], and the recently started PILOTING project aims at further developing these technologies to help them reach the commercialization stage [20].

Nowadays, there are three main approaches for infrastructure contact inspection. One of them consists of using a multirotor which can stick firmly to the bridge beams and place a reflector prism in contact with the bridge surface to measure beam deformation using a robotic Total Station on ground, which can measure the position of the prism with millimeter accuracy [21]. The main advantage of this approach is that the measurements can achieve the required accuracy for structural assessment since the robot is completely still while the inspection measurements are collected. However, the robot is not able to move over the bridge surface once it is touching it, making it very difficult or even impossible to reach the location of a specific defect in a second inspection. The second approach uses aerial manipulators with a robotic arm placed on the upper part of the multirotor to reach the inspection area while flying [22]. This solution opens the spectrum of applications that can be accomplished because the end effector of the manipulator can be designed for a specific use case, such as sensor installation [23], hammering tasks [24], deflection measurements [25], and others. The third approach is about using a morphing platform or a more complex robot with the capability of moving along the infrastructure, maintaining the contact with the surface [26,27]. Nevertheless, although the last two approaches seem to be more versatile, the required positioning accuracy of the robot and the requirements in the measurement collection process are, again, very difficult to reach while flying and sensor installation usually requires a controlled force while the glue is curing to grant the adhesion of the device to the surface. These are challenging applications in which the robot must fight the environmental disturbances, like wind and aerodynamic effects among others, while the end effector of the robotic arm is applying a constant and controlled force.

On the other hand, the aerial robot in [25] and most aerial manipulators [3,4,5,6,7,8,9,10] use standard multirotor configurations with 4, 6, or 8 co-planar propellers. They are, therefore, under-actuated robots that need to tilt to move or apply forces in the horizontal plane. Several designs have been developed in the literature to overcome this limitation and achieve full range of motion in the 6 DOF, which are generally known as multi-directional thrust multirotors. A first group have the motors fixed to the frame with different non-parallel axes, and can achieve full range multi-directional motion without any other actuation [28,29,30,31]. Other designs are able to rotate their rotors about the axis of the individual multirotor arm [32,33,34,35] or with bi-axial tilting mechanisms [36,37], or rotate frame sections [38], although they have the penalty of the added weight of the tilting mechanism and actuation.

The multi-directional multirotor designs have also been combined with a robotic arm with few additional degrees of freedom to reach the desired position with the end-effector for pipe inspection in oil and gas plants [39,40]. Although they have not been used before for infrastructure inspection, fully actuated platforms have good characteristics for accurately positioning the arm end-effector and applying forces in confined spaces as tunnels and below the bridge deck.

Aerodynamic effects can arise also in these kinds of scenarios and applications, in which the aerial robot is required to operate in the surrounding of the infrastructure. Previous works have shown how the aerodynamic ceiling effect can change the performance of a multirotor when it approaches a surface from the underside [41,42] and usually requires using a specific controller [21,43]. However, in this specific work, the system will operate far enough from the infrastructure not to be affected by this aerodynamic disturbance.

**Figure 1:**
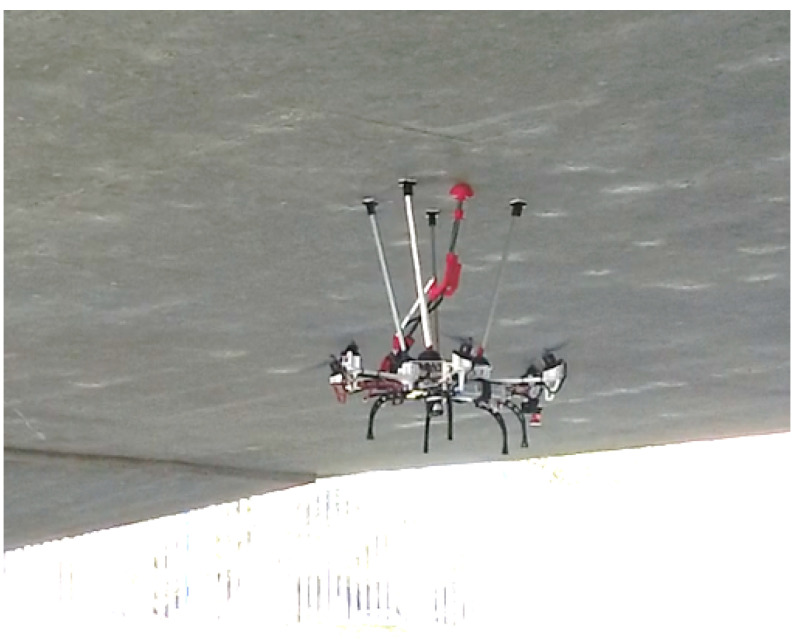
Fully-actuated aerial robot during an inspection by contact operation.

This paper presents the design of an aerial manipulator (see Figure 1) based on a fully actuated hexarotor with tilted rotors and a special arm placed at the top for contact inspection of bridges and tunnels. It describes a new prototype of aerial manipulator including the aerial platform, the robotic arm, and a specific “docking gear” focused on bridge inspection. The aerial platform uses a standard multirotor frame adapted to be fully actuated. The robotic arm has compliant joints that allow it to adapt and absorb contact forces, and has sensors to measure joint rotation and deformation. A custom sensing device has been included inside the arm to minimize its weight and size. Finally, the device called “docking gear” acts as a landing gear mounted on the top of the aerial platform which helps to establish the contact between the bridge and the aerial robot and maintain a security distance avoiding also being affected by the aerodynamic ceiling effect. In this way, it significantly increases the positioning accuracy of the end effector of the arm and facilitates its force control because the robot is not moving while inspecting. Moreover, the docking gear also improves the safety conditions preventing the propellers from hitting the structure and, thanks to their material in the contact interface, it helps to precisely maintain the position during the operation. These two aspects are considered by the end-users of this technology as mandatory and essential for proper commercial exploitation in bridge maintenance programs. Since the use case of this robot imposes flying very close to the infrastructure inspected, a localization system to operate in GNSS denied environments is also presented using a robotic total station and a commercial camera which is running a VSLAM algorithm [44]. Such algorithm has been optimized by Intel and it runs in an internal processor of the camera, fusing data from an IMU and stereoscopic vision [45]. The modeling and control techniques used in the robot are also described and validated through outdoors experiments.

The rest of paper is organized as follows: Section 2 presents a description of the global system including details related to the hardware and software of the final solution and the decision criteria. Section 3 presents the localization system to operate in a GNSS denied environment and in a time consistent reference frame. Section 4 describes the dynamic model of both the multirotor and the robotic arm followed by the control techniques implemented in these subsystems. Section 5 shows the experiments used to validate the system and its capabilities and, finally, conclusions and future works are presented in Section 6.

## 2. System Description

The aerial part of the system proposed is composed of three main subsystems: the fully-actuated aerial platform, the robotic arm (both specifically designed for bridge inspection applications), and the localization system. On the other hand, the ground part consists of three subsystems: a ground control station running on a PC with MATLAB/Simulink, a robotic total station which is used as the inspection sensor, and it is in charge of providing the position estimation of the aerial platform in case of the flight is carried out in a GNSS-denied environment, a standard router to establish the link between the autopilot on-board and the ground control system (GCS) which is also the robotic total station server. All these components as well as the software/hardware architecture are described in detail in this section.

### 2.1. Tilted Rotor Design

As it was mentioned in the Introduction, standard hexarotors with co-planar propellers are under-actuated platforms in terms of the force that they can exert and the maneuvers that they can perform. However, both characteristics are essential in the field of aerial contact-inspection robotics and give a chance for the application of a fully-actuated platform. The simplest multirotor considered as a fully-actuated platform consists of a hexarotor with tilted propellers to exert a full vector of forces and torques including lateral forces, unlike a co-planar hexarotor which is only able to develop four control actions, the vertical force, and the three torques. In other words, a tilted hexarotor has not only more control capability, but also an enhanced performance followed by the ability to exert forces in the horizontal plane without tilting the platform, which has a priceless value in aerial manipulation applications.

**Figure 2:**
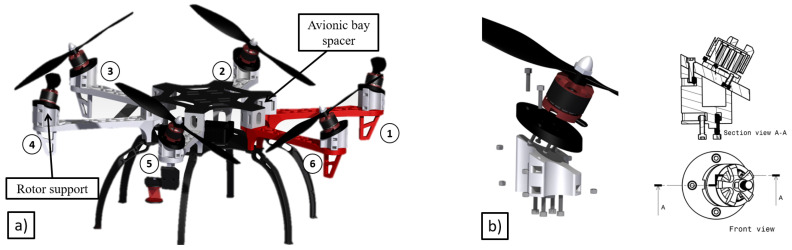
Fully-actuated platform. (**a**) 3D concept; (**b**) rotor support detail.

The prototype presented in this work (see Figure 2a) has been built customizing a DJI F550, a commercial frame available in the market, and the selected motorization is the combination of the rotors DJI 2312E rotors with the DJI 9 × 4.5 in propellers and the XRotor 40A ESCs as propulsive system.

The main modifications to the commercial frame are: spacers to increase the space in the avionics bay to be able to integrate the autopilot board and supports to tilt the rotors and provide the ability to exert the above-mentioned horizontal forces. In order to facilitate the installation, these supports are formed by two separated pieces. The left side in Figure 2b shows an explodsion of both parts and the screws necessary for its union. The white part shown is the one in charge of making the inclination of the rotor. This piece is screwed with the frame using four screws and four nuts, which are placed in four side cavities of the piece. The black part is in charge of making the union between the motor and the white piece. To do this, first, the black piece is screwed to the motor, and finally the black piece to the white piece. Due to this frame being widely used in the research community, the authors have decided to share the designed parts to transform this platform in a multirotor with tilted propellers (Stl files in https://hdvirtual.us.es/discovirt/index.php/s/YPGbxt9Lz96MSQ3). This link also hosts the spacer part designed to increase the avionic bay of the DJI frame. All the modifications to the original design of the DJI frame have been mainly printed in ABS.

**Figure 3:**
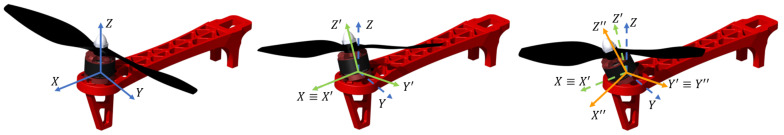
Sequence of the propeller rotation.

Following the process described in [46] and for the purpose of minimizing the control effort, the global tilt angle of the propellers has been decomposed in two rotations around a local reference frame linked to the rotor. The sequence of this rotation is shown in Figure 3 and consisting of a first rotation of an angle $\alpha_{i}$ around the $X_{i}$-axis, and a second one of $\beta_{i}$ in the $Y_{i}^{{}^{\prime }}$-axis, where the index *i* represents the *i*-rotor being i=1, the front left rotor and following a counter-clockwise direction for the others (see Figure 2a and Figure 3). Moreover, in order to simplify the design, it has been imposed that the $\forall i∥\alpha_{i}∥=\alpha =∥\beta_{i}∥=\beta =20^{\circ }$, and their signs are such that $\alpha_{i}=-\alpha$ and $\beta_{i}=\beta$ when i=2n+1 where n=0,1,2 and $\alpha_{i}=\alpha$ and $\beta_{i}=-\beta$ when i=2n being n=1,2,3, as it is summarized in Table 1. The value of $20^{\circ }$ is justified due to this inclination does not compromise the lift of the multirotor and guarantees enough lateral forces for inspection by contact operations.

In order to analyze the fully-actuation of the platform, we study the forces on the *x*-axis and *y*-axis that the platform is able to generate, without modifying its orientation, depending on the angles of inclination of the motors α and β. To obtain these work regions, the optimization problem (1) has been solved for different values of α and β, where *F* is the modulus of the force exerted in the direction $\vec{v}$, *m* is the mass of the platform, *g* is the acceleration of gravity, M(α,β) is the mixer matrix, presented in Section 4, and $\omega_{min}$ and $\omega_{max}$ are the maximum and minimum speeds of the motors:

(1) $$\begin{array}{cccc} \; & \underset{\omega^{2}\in R^{6}}{\mathrm{maximize}}\; & \; & F\\ \; & \mathrm{subject}\;\mathrm{to}\; & \; & M\left(\alpha ,\beta \right)\omega^{2}=\left[F\vec{v}+mg\vec{z};0;0;0\right],\\ \; & \; & \; & \omega_{min}^{2}<\omega^{2}<\omega_{max}^{2}. \end{array}$$

**Figure 4:**
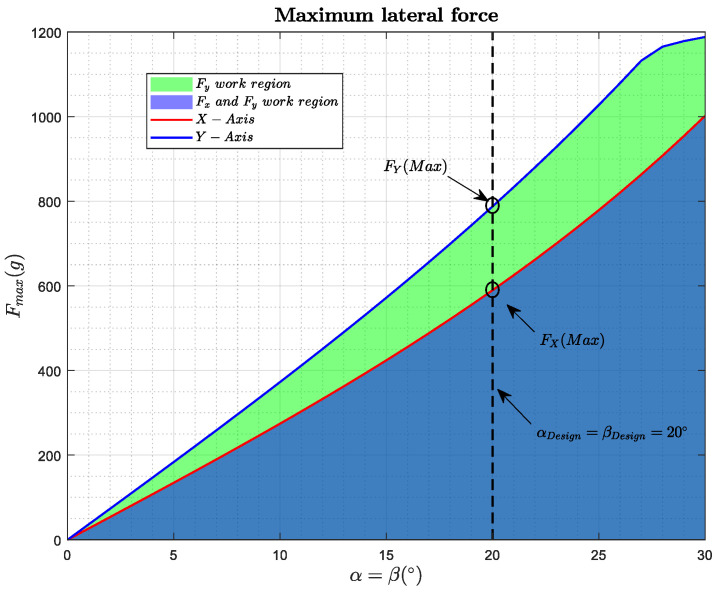
Maximum lateral force achievable by the platform.

Figure 4 shows the results of the optimization problem for the *x*-axis and *y*-axis. It can be seen how the forces can be generated whenever we are under the blue curve, for the *y*-axis, and the red curve for the *x*-axis. At the moment that we exceed these curves, we will not be able to generate these lateral forces maintaining a null inclination angle.

In addition, to continue to see the limits of the platform, the maximum angle of inclination of the platform in hover has been studied. For this, the optimization problem (2) has been solved, where θ is the inclination angle around the v−axis, $R_{v}$ is the rotation matrix around the *v*-axis, *m* is the mass of the platform, *g* is the acceleration of gravity, M(α,β) is the mixer matrix, and $w_{min}$ and $w_{max}$ are the maximum and minimum speeds of the motors:

(2) $$\begin{array}{cccc} \; & \underset{\omega^{2}\in R^{6}}{\mathrm{maximize}}\; & \; & \theta \\ \; & \mathrm{subject}\;\mathrm{to}\; & \; & M\left(\alpha ,\beta \right)\omega^{2}=\left[R_{v}\left(\theta \right)mg\vec{z};0;0;0\right],\\ \; & \; & \; & \omega_{min}^{2}<\omega^{2}<\omega_{max}^{2}. \end{array}$$

**Figure 5:**
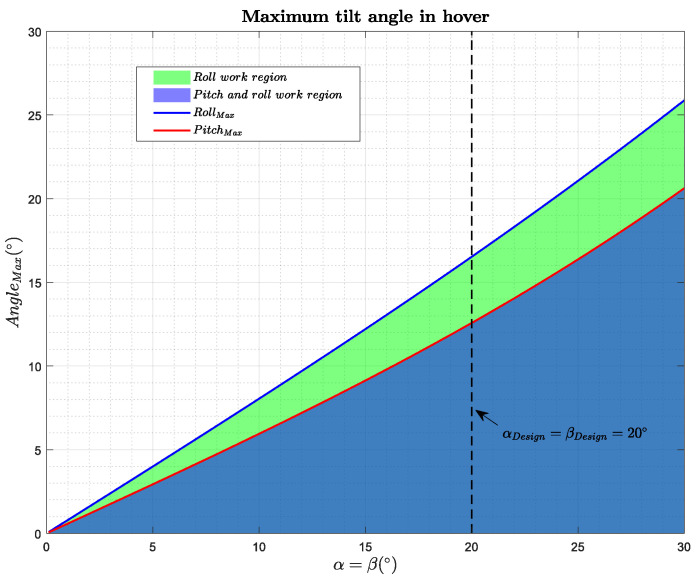
Maximum tilt angle in hover achievable by the platform.

Figure 5 shows the maximum roll and pitch angles in a hovering maneuver. Below these curves is the working region, i.e., the region where our platform is still a full platform.

The main features of the fully-actuated platform designed are shown in Table 2. Note that the weight presented in this table is the minimum take off weight including batteries and avionics and that the *Maximum achievable lateral force* are the maximum forces exerted by the platform limiting the thrust of the motors to the 90% of their maximum thrust.

### 2.2. Arm Design

**Figure 6:**
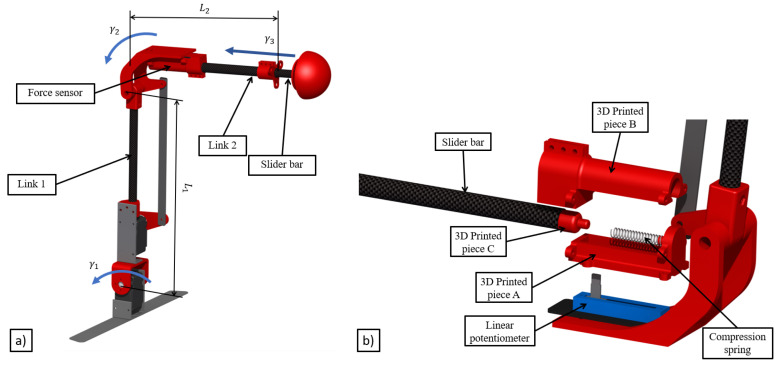
Lightweight robotic arm. (**a**) 3D concept; (**b**) force sensor detail.

**Figure 7:**
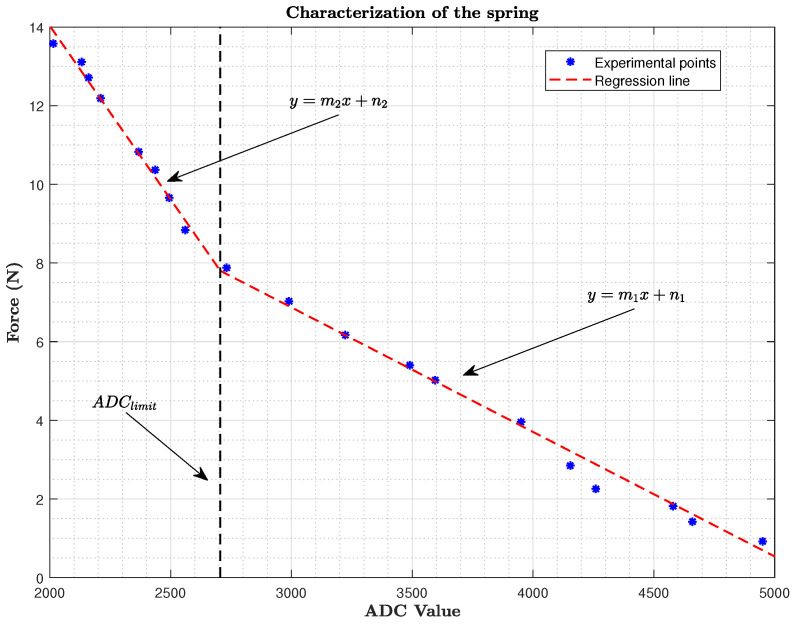
Spring experimental characterization.

The aerial platform presented in this paper is equipped with a 3DoF lightweight robotic arm (see Figure 6a), which has been designed minimizing the inertia as much as possible. In order to achieve this objective, light materials like carbon fiber links, ABS, and aluminum have been used. The servo motors have been installed as close as possible to the base of the arm to minimize the changes in the mass distribution due to the movement of the arm links. Moreover, it is equipped with a force sensor to accomplish the inspection operations [47], such as installing a sensor or collecting data to monitor the bridge health. The sensor consists of a linear potentiometer and a compression spring installed in the third joint which is therefore a compliant joint (Figure 7). In this way, the arm is able to measure the contact force that it is available also to the autopilot which is an integrated controller that considers the dynamic behavior of both the aerial platform and the robotic arm.

Figure 6a shows the three degrees of freedom of the lightweight robotic arm, where $\gamma_{1}$ is the shoulder pitch angle, $\gamma_{2}$ is the elbow pitch angle, and $\gamma_{3}$ is the slider bar displacement.

The force sensor is composed of four components: a linear potentiometer, a compression spring, a carbon fiber bar, and 3D printed pieces (see Figure 6b). The linear potentiometer is fixed on the elbow of the arm by the plastic piece A. This part also holds the spring at one end. The plastic piece B encapsulates the whole sensor and holds the link B of the arm, inside which the carbon fiber bar slides. Finally, the carbon fiber bar has a plastic piece C at its end, which facilitates the contact with the spring.

The stiffness of the spring has been characterized experimentally. Different values of the compression forces have been applied to the spring through a variable weight, and the sensor value measured at the ADC port has been recorded. With all the data, a two-section piece-wise linear approximation has been done (Figure 7) and the parameters can be seen in Table 3. Finally, Table 4 shows the main features of the robotic arm.

### 2.3. Final Prototype Integration

**Figure 8:**
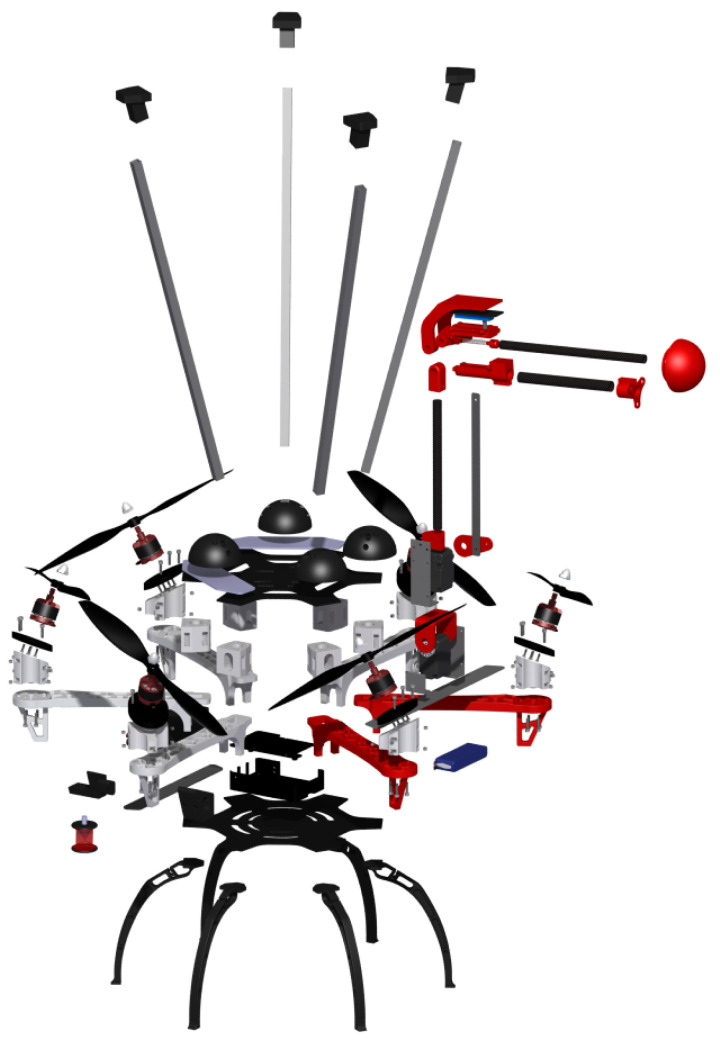
Exploded view of the system.

The final integrated system (see Figure 8) with the aerial platform and the robotic arm has been completed with the installation of the aforementioned “docking gear”, which acts as a landing gear, installed on the top of the aerial platform, made of aluminum and ABS parts. The main function of this device is to facilitate the inspection decoupling the controller of the aerial platform during the inspection phase from the inspection operation guaranteeing that the contact is safe and stable. The device has been lined with a rubbery material which prevents horizontal movements while inspecting. On the other hand, the robotic arm has been installed between two rotors in order to increase the arm workspace, using a part manufactured in aluminum. Although the robotic arm has not been mounted in the geometric center of the UAV, the batteries have been placed to guarantee that the center of the mass (CoM) of the entire system matches this point.

From the application point of view, it is important to remark that, although the system has already been tested inspecting bridge beams, it has also been designed with the capabilities of inspecting vertical structures such as pillars or walls. This is possible because the position of the robotic arm allows it to reach positions beyond the propeller’s workspace.

### 2.4. On-Board Avionics

**Figure 9:**
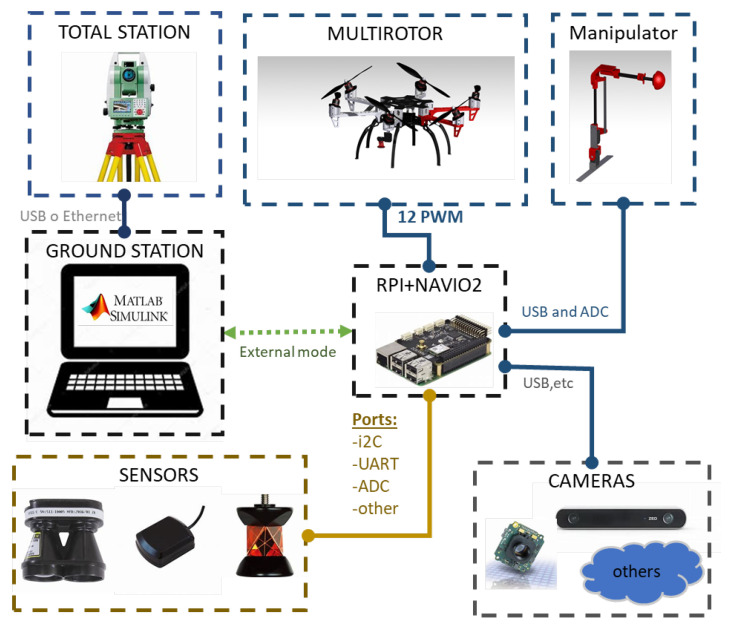
Hardware architecture.

The on-board avionics consists of a custom autopilot code running in a Raspberry Pi 3 Model B connected to the Emlid’s sensors shield Navio2. The aerial platform and the Ground Control Station (GCS) are connected by Wi-Fi Technology. A range sensor model SF11/C has also been mounted on the platform. This prototype has been equipped with a multi-sensor localization system using two kinds of sensors, a laser sensor on the ground with a reflective target on-board, and a VSLAM sensor on-board. The first one consists of a Leica MS50 and a GRZ101 360${}^{\circ }$ reflector prism on-board. This Leica MS50 is a robotic total station which was defined in [48] as “a robotized sensor used to measure distances with laser technology which can measure the distance to any surface or a reflector prism using a highly accurate laser system”, in this case, we are using the aforementioned reflector prism to improve the measurements and facilitate the tracking. The second sensor on-board will be an Intel Realsense T265 tracking camera, Santa Clara, CA, USA, which is a stand-alone solution for simultaneous localization and mapping. The measurements of the total station are communicated by a Wi-Fi link with the autopilot and then those are integrated with the camera estimation using an extended Kalman filter running in the Raspberry Pi. After that, the output of this Kalman filter is used as feedback in the position control loop. In this way, it is possible to carry out autonomous flights in GNSS denied environments such as underneath bridges or tunnels. Section 3 explains in detail the fusion algorithm as well as the alignment method and data preparation stage, it also emphasizes the advantages of using this multi-sensor approach which mixes and absolute localization system (total station) with a local one (tracking camera). Finally, Figure 9 shows a scheme of the hardware system mounted in this prototype.

The aerial robot controller has been developed using our GRVC autopilot tool (https://github.com/AEnrique/GRVCAutopilot) [49]. This tool has been developed in the University of Seville and designed to be used inside a Raspberry Pi^®^ 3B with a Navio^®^2 sensor shield. The autopilot allows the implementation of control strategies at low and high levels using MATLAB/Simulink, as well as the easy integration of new sensors and data monitoring.

## 3. Localization System

An aerial robot flying close to or under the bridge deck will have in general poor GNSS satellite visibility or no visibility at all. Thus, for this application, the aerial robot needs a reliable localization system that does not use the measurements of the GNSS receivers.

Moreover, although there are several solutions that aim to solve this localization problem in a GNSS-denied environment, those usually consist of algorithms that generate a local reference frame each time they are executed, like the classical VSLAM [44], LOAM [50], and other SLAM algorithms [51]. However, the use case of infrastructure inspection imposes other unavoidable requirements for the localization system. On the one hand, it is necessary to define a common time-consistent reference frame to be able to localize the defects once they are detected and reach their location in different flights and days to assess their evolution over time. This reference frame must be independent of the sensors on-board and, preferably, it should be linked to the physical infrastructure. In this way, the defects as well as the flight plan and others will be referenced in a common frame and will not depend on the initial position and orientation of the platform which may vary from one flight to another.

### 3.1. Multi-Sensor Localization for Bridge Inspection

The proposed solution consists of creating a reference frame using a Leica MS50 robotic total station to solve the time-consistent reference frame because, with the Leica software, it is possible to measure and store the position of non-deformable points of the terrain as a reference of this time-consistent reference frame. Then, in a second inspection which could be carried out deploying the Leica in a different location or even inspecting in a different day, those reference points could be measured again on a local axis, but, using the least squared method, and the total station triangulates its position and axes in the original time-consistent reference frame. However, despite in a previous work [48], this device has been used to directly feedback the position control loop in the autopilot, the total station has drawbacks when it is used as a stand-alone positioning sensor. First, the sample rate is variable from 0 up to 20 Hz. Second, it is necessary to keep the reflective target mounted on the platform in a direct line of sight with the total station. In addition, finally, since the total station must be deployed on the ground, it requires using a wireless link between the robot and the Leica server, and, if the communication fails, the robot will lose the positioning measurements and lead to a crash.

For those reasons, it was proposed to use a multi-sensor solution, which should be able to converge to the axis created by the total station with on-board sensors that do not depend on a wireless link and work in a more stable way. In this case, the measurements provided by the robotic total station will be combined with the ones provided by an Intel RealSense T265, a dual fish-eye lens camera that is running a VSLAM algorithm out of the box. In the same way, this solution could be applied to other SLAM or LOAM techniques. Those algorithms usually work creating their own local reference frame which depends on the initial position and orientation of the aerial platform, it being necessary to define a calibration protocol to align them to the common reference frame established by the robotic total station.

In this case, the calibration protocol consists of collecting data while moving the aerial platform manually or in a first security flight while the different algorithms are running in parallel. After that, it is necessary to calculate the deviation between the different frames and the physical frame, and, using a numerical method like the least square fitting algorithm, it is possible to obtain the transformation of each local inertial reference system to the common one.

Once we have the set of data, the first task to do is to make it uniform, that is to say, since the sensors have different sample rate, the length of the data vector will not be the same for each sensor, and it is necessary to be the same for the sensor fusion algorithm. We shall use linear interpolation presented in [52].

Now, we can introduce the alignment process. We define the following transformation equation, which transforms a point from the camera frame to the total station frame:

(3) $$\hat{x}=Tx+P_{0},$$

where *x* is the position given by the VSLAM algorithm of the camera, $\hat{x}$ the position given by the total station, *T* the rotation matrix from the camera frame to the total station frame, and $P_{0}$ the multirotor initial position. We have to use all the points prepared previously to conform an over-determined linear equation system and to obtain the nine components of *T* ($t_{ij}$) and the three components of $P_{0}$ ($p_{i}$). Finally, the prepared equation system results as follows:

(4) $$\left(\begin{array}{c} {\hat{x}}_{1}\\ {\hat{x}}_{2}\\ \vdots \\ {\hat{x}}_{n} \end{array}\right)={\left(\begin{array}{cccccccccc} x_{1} & \; & \; & x_{2} & \; & \; & \; & x_{n} & \;\\ \; & x_{1} & \; & \; & x_{2} & \; & \cdots & \; & x_{n} & \;\\ \; & \; & x_{1} & \; & \; & x_{2} & \; & \; & \; & x_{n}\\ \; & I_{3} & \; & \; & I_{3} & \; & \cdots & \; & I_{3} & \; \end{array}\right)}^{T}u,$$

where $I_{3}$ is the three-dimensional identity matrix, ${\left(\cdot \right)}^{T}$ the transposition operator, and *n* the number of total points after interpolation. The blank spaces of the above matrix are supposed to be 0 and *u* is the unknowns vector

(5) $$u={\left(\begin{array}{cccccccccccc} t_{11} & t_{12} & t_{13} & t_{21} & t_{22} & t_{23} & t_{31} & t_{32} & t_{33} & p_{1} & p_{2} & p_{3} \end{array}\right)}^{T}.$$

This over-determined equation system can be solved using a least square fitting algorithm implemented in MATLAB or using any C++ library such as *Armadillo* (https://gitlab.com/conradsnicta/armadillo-code) to solve it on-board.

**Figure 10:**
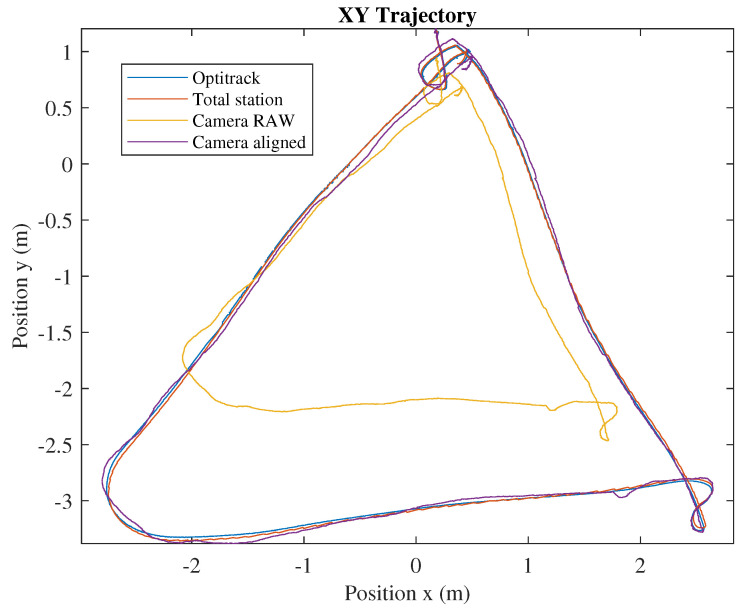
Camera alignment.

Figure 10 shows an example of how this method works adjusting the reference system of the RealSense T265 and the Leica MS50. The XY trajectory of a flying platform manually piloted has been represented. It has also been represented the trajectory seen by the Optitrack of the University of Seville, which is a high precision motion capture system that has been used as ground truth. In yellow, the raw trajectory calculated by the tracking camera is shown. It can be clearly observed that, by default, it suffers scale, alignment, and position errors. However, it is clearly shown that the aforementioned calibration protocol improves the results of the camera and automatically transforms their measurements for the absolute reference frame created by the total station. The results of the camera after performing the calibration are represented in purple.

Now, it is possible to employ all the data to get an accurate estimate of the multirotor position using the Kalman filter [53,54]. We first define the following state vector at instant *k*, where the three first components are the vehicle position and the last three ones are its velocity

(6) $$x_{k}={\left(\begin{array}{cccccc} y_{1} & y_{2} & y_{3} & {\dot{y}}_{1} & {\dot{y}}_{2} & {\dot{y}}_{3} \end{array}\right)}_{k}^{T}.$$

The linear difference equation that governs the vehicle state is:

(7) $$\begin{array}{cc} x_{k} & =Ax_{k-1}+w_{k-1}, \end{array}$$

and a measurement is given by

(8) $$z_{k}=Hx_{k}+v_{k}.$$

Here, *w* and *v* are random variables representing the process and the sensors noise, respectively. They are assumed to be white and with normal probability distribution:

(9) $$\begin{array}{c} p(w)∼N(0,Q), \end{array}$$

(10) $$\begin{array}{c} p(v)∼N(0,R). \end{array}$$

Matrix *A* is built using kinematics equations, integrating velocity to obtain the position:

(11) $$A=\left(\begin{array}{cc} I_{3} & T\cdot I_{3}\\ 0_{3\times 3} & I_{3} \end{array}\right),$$

where $0_{3\times 3}$ is a 3 by 3 zero matrix and *T* the elapsed time from k−1 to *k*. *H* relates the state with the sensors measurements, since our sensors provide us with the position and velocity directly; this matrix is the identity matrix.

The first stage of Kalman filter is prediction. We will predict the state vector and its covariance matrix *P*

(12) $$\begin{array}{cc} {\hat{x}}_{k}^{-} & =A{\hat{x}}_{k-1}, \end{array}$$

(13) $$\begin{array}{cc} P_{k}^{-} & =AP_{k-1}A^{T}+Q. \end{array}$$

The second stage of Kalman filter is to correct the state and covariance matrix using sensors data

(14) $$\begin{array}{cc} K_{k} & =P_{k}^{-}H^{T}{\left(H_{k}P_{k}^{-}H^{T}+R\right)}^{-1}, \end{array}$$

(15) $$\begin{array}{cc} {\hat{x}}_{k} & ={\hat{x}}_{k}^{-}+K_{k}\left(z_{k}-H{\hat{x}}_{k}^{-}\right), \end{array}$$

(16) $$\begin{array}{cc} P_{k} & =\left(I-K_{k}H\right)P_{k}^{-}. \end{array}$$

In order to tune the Kalman Filter, due to it being hard to estimate the measurements and process noise (*R* and *Q*), we employed a heuristic method, changing the parameters manually until the filter provided an acceptable response. For the sake of simplicity, the parameters were not supposed correlated, that is to say, the identity matrix multiplied by a certain gain. The resulting parameters are $Q=2\cdot 10^{-6}\times I_{6}$ (Process noise covariance), $R_{ts}=5\cdot 10^{-3}\times I_{6}$ (Total Station measurement covariance), and $R_{c}=1\cdot 10^{-2}\times I_{6}$ (Camera VSLAM measurement covariance).

We can summarize the whole process in the following steps:

1. Align sensors to the common reference frame:
  (a) Move the aerial platform manually or in a first safe flight and record the measurements from the sensors.
  (b) Interpolate the collected sensor data to have the same length.
  (c) Solve the over-determined equation system using a least square fitting algorithm to obtain *T* and $P_{0}$.
2. Apply the Kalman filter to obtain an accurate estimate of the platform position.

**Figure 11:**
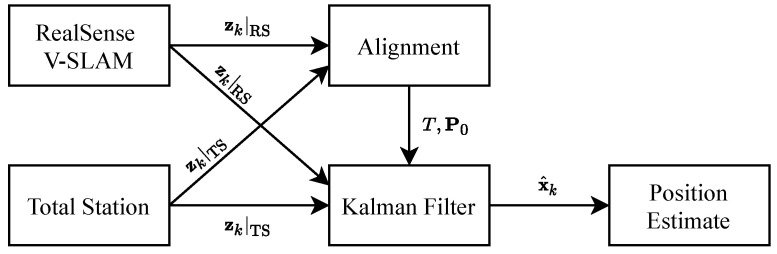
Positioning system architecture.

Figure 11 shows the architecture of the positioning system.

### 3.2. Experimental Results

**Figure 12:**
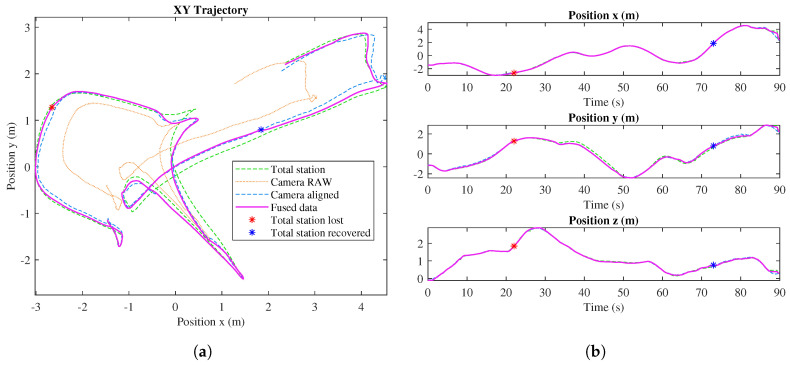
Fused trajectory using Kalman Filter (**a**) and separated coordinates *x*, *y*, and *z* (**b**).

Figure 12 shows the results of the localization system in an outdoor experiment. In this experiment, we could not use Optitrack as ground truth because it is not available in our outdoor experimental field; however, in Figure 10, we can see that Total Station has enough accuracy as ground truth and can be used as a valid reference system in outdoor applications. The UAV starts the flight at (−1.5, −1). The pink line represents the outcome of the implemented Kalman filter algorithm, fusing the data from the aligned tracking camera and the robotic total station. In this test, a failure in the total station was simulated (red asterisk), even though it is shown that the algorithm continues estimating the position just using the tracking camera. When the total station is recovered at 73 s (blue asterisk), the accumulated error between the position estimated with the VSLAM algorithm and the real position in the Leica reference system is 20.5 cm, and, after that, the filter uses both the total station and tracking camera measurements again.

Finally, we conclude that, for getting the position of the aerial platform, we will use the method previously presented because it solves the sensor alignment problem, it provides a time-consistent reference frame, it is GNSS-free, and it fuses all the available sensor measurements, getting a precise localization algorithm that is robust against sensor failures. Additionally to this approach, the next efforts will be devoted to extend this methodology including a 3D LIDAR.

## 4. Modeling and Control

### 4.1. Modeling

**Figure 13:**
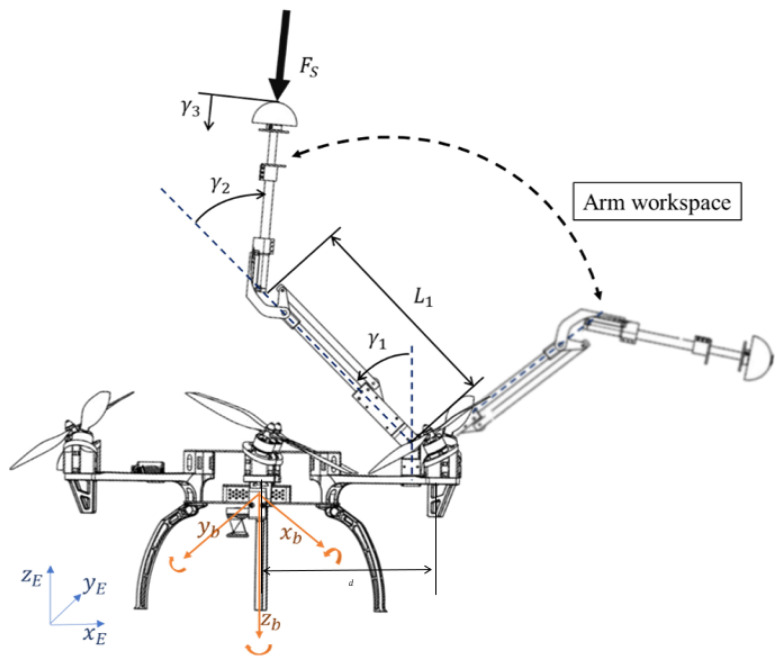
Joint angles definition.

The dynamic model for a multirotor and a 3-link manipulator arm can be obtained using the Euler–Lagrange formulation. Defining the state vector as $\xi ={\left[p\;\eta \;\gamma \right]}^{T}$, where p=[xyz] is the position of the multirotor center of gravity in Cartesian coordinates in the Earth fixed frame $\left\{E\right\}$, η=[ϕθψ] that represents the Euler angles of the aerial platform attitude, commonly known as roll, pitch, and yaw in the multirotor base or body frame $\left\{B\right\}$ that is attached to the IMU of the autopilot, $\gamma =[\gamma_{1}\;\gamma_{2}\;\gamma_{3}]$ is the vector which contains the joint angles of the robotic arm, $\gamma_{1}$ and $\gamma_{2}$ are the active links while $\gamma_{3}$ is the passive one which acts as a force sensor during the contact. These variables are illustrated in Figure 13.

Thus, the dynamic equations of the aerial manipulator can be obtained using the Euler– Lagrange equations:

(17) $$\frac{d}{dt}\frac{\partial L}{\partial {\dot{\xi }}_{i}}-\frac{\partial L}{\partial \xi_{i}}=F+F_{C}$$

where L is the Lagrangian of the system defined as L=T−V, where $T=1/2\sum_{k=1}^{N}m_{k}v_{k}^{2}$ is the total kinetic energy of the system and *V* is the potential energy.

In the Euler–Lagrange equation, F is the vector of forces and torques generated by the actuators, $F_{C}$ includes the contact forces that can be estimated with the sensor installed in the end effector and they are mainly a function of the passive joint, $F_{C}∼F_{C}\left(\gamma_{3}\right)$. Thus, the dynamic model of the full system can be written in compact matrix form as:

(18) $$M\left(\xi \right)\ddot{\xi }+C\left(\xi ,\dot{\xi }\right)\dot{\xi }+G\left(\xi \right)=F\left(\alpha ,\beta \right)+F_{C}\left(\xi \right)+F_{ext}$$

where M(ξ) is the inertia matrix, $C(\xi ,\dot{\xi })$ represents the Coriolis and centrifugal terms, the gravitational forces that are represented by G(ξ), which depends on the state of the system due to the movements of the aerial manipulator, change the position of the center of gravity of the platform. On the other hand, F(α,β) collects the forces and torques of the rotors and active joints. $F_{C}\left(\xi \right)$ models the contact forces and $F_{ext}$ includes external forces and torques which are not known or modeled like the ones produced by the wind or the environment.

In summary, these forces can be expressed as:

(19) $$F\left(\alpha ,\beta \right)=\left[\begin{array}{c} F_{w}\left(\alpha ,\beta \right)\\ F_{a} \end{array}\right]$$

where $F_{w}\left(\alpha ,\beta \right)$ is the vector of forces generated by the rotors, and $F_{a}$ are the torques generated by the arm joint servos, as follows: $C_{T}$ being the thrust coefficient of the rotors, *L* the distance from the CoM to the rotor position, and $C_{f}$ the drag coefficient.

Matrices $A_{3\times 6}$ and $G_{3\times 6}$ in (20) have the form:

$$F_{w}\left(\alpha ,\beta \right)=N\left(\alpha ,\beta \right){\left[\begin{array}{ccc} \omega_{1}^{2} & \cdots & \omega_{6}^{2} \end{array}\right]}^{T}=\left[\begin{array}{c} A_{3\times 6}C_{T}\\ G_{3\times 6}LC_{T}+H_{3\times 6}C_{f} \end{array}\right]$$

(20) $$F_{a}={\left[\begin{array}{ccc} \tau_{1} & \tau_{2} & 0 \end{array}\right]}^{T}$$

(21) $$A_{3\times 6}=\left[\begin{array}{cccccc} \frac{\sqrt{3}}{2}s\beta -\frac{s\alpha c\beta }{2} & s\alpha c\beta & -\frac{\sqrt{3}}{2}s\beta -\frac{s\alpha c\beta }{2} & \frac{\sqrt{3}}{2}s\beta -\frac{s\alpha c\beta }{2} & s\alpha c\beta & -\frac{\sqrt{3}}{2}s\beta -\frac{s\alpha c\beta }{2}\\ \frac{s\beta }{2}+\frac{\sqrt{3}}{2}s\alpha c\beta & -s\beta & \frac{s\beta }{2}+\frac{\sqrt{3}}{2}s\alpha c\beta & \frac{s\beta }{2}+\frac{\sqrt{3}}{2}s\alpha c\beta & -s\beta & \frac{s\beta }{2}-\frac{\sqrt{3}}{2}s\alpha c\beta \\ c\alpha c\beta & c\alpha c\beta & c\alpha c\beta & c\alpha c\beta & c\alpha c\beta & c\alpha c\beta \end{array}\right]$$

(22) $$G_{3\times 6}=\left[\begin{array}{cccccc} \frac{c\alpha c\beta }{2} & c\alpha c\beta & \frac{c\alpha c\beta }{2} & -\frac{c\alpha c\beta }{2} & -c\alpha c\beta & -\frac{c\alpha c\beta }{2}\\ \frac{\sqrt{3}}{2}c\alpha s\beta & 0 & \frac{\sqrt{3}}{2}c\alpha s\beta & \frac{\sqrt{3}}{2}c\alpha s\beta & 0 & -\frac{\sqrt{3}}{2}c\alpha s\beta \\ s\alpha c\beta & -s\alpha c\beta & c\alpha c\beta & -s\alpha c\beta & c\alpha c\beta & -s\alpha c\beta \end{array}\right]$$

In addition, the $H_{3\times 6}$ matrix (20) can be obtained from $A_{3\times 6}$, changing the sign of the first, third, and fifth columns.

Relative to the force measured through the sensor installed in the end-effector, $F_{C}$ could be written as:

(23) $$F_{C}\left(\xi \right)=\left[\begin{array}{cc} R^{EB} & 0\\ 0 & I \end{array}\right]$$

(24) $$\left[\begin{array}{c} -F_{s}\sin\left(\gamma_{1}-\gamma_{2}\right)\\ 0\\ -F_{s}\cos\left(\gamma_{1}-\gamma_{2}\right)\\ 0\\ F_{s}d\cos\left(\gamma_{1}-\gamma_{2}\right)-F_{s}L_{1}\sin\left(\gamma_{2}\right)\\ 0\\ F_{s}L_{1}\sin\left(\gamma_{2}\right)\\ 0\\ F_{s} \end{array}\right]$$

$F_{s}$ being the force measured with the sensor which is assumed as an input data such that $F_{s}=K_{s}\gamma_{3}$, $K_{s}$ is the spring stiffness constant and *d* is the distance from the CoM to the point in which the arm is attached, and $R^{EB}\in SO\left(3\right)$ is the rotation matrix which transforms a vector in body frame $\left\{B\right\}$ to the inertial earth fixed frame $\left\{E\right\}$. These models of forces and torques are essential to provide the fully actuated capabilities and also to take it into account to guarantee the safety and stability of the aerial platform during the contact phase. This model is validated by comparing model prediction with real flight data in Section 5.1.

### 4.2. Control Scheme

The design of the aerial manipulator controller combines the backstepping approach in [55] with an open-loop adaption of the inertia matrix to improve the performance of the aerial platform and the manipulator arm [56]. After estimating the six independent inertia values for each of the manipulator settings and its validation (See Section 5), the application of certainty-equivalence principle is immediate. The demonstration of its suitability for a standard multirotor with a robotic arm has been proved in [56,57]. In this section, the previous approach has been adapted to a fully actuated platform with a robotic sensorized arm.

One of the major advantages of this technique is that it allows the coupled control of the full system dynamics and the external forces induced by surface contact and the arm movements. Moreover, thanks to the linear potentiometer placed at the end effector (see Figure 6b), the external forces during the contact can be estimated and the torques applied in each arm joint, due to the contact force, can be calculated from (24). The estimation of these forces and torques allows the implementation of a controller to track trajectories on a structure surface applying the desired force needed to carry out measurements of deflections in a beam, the inspection of cracks using an ultrasonic sensor or sensor placement in the infrastructure. On the other hand, due to the servo actuators used in the robotic arm, the controller outputs, $U_{\gamma }$, must be mapped to desired joints inputs, $\gamma_{d}$. This mapping has been generated thanks to a standard procedure of system identification.

**Figure 14:**
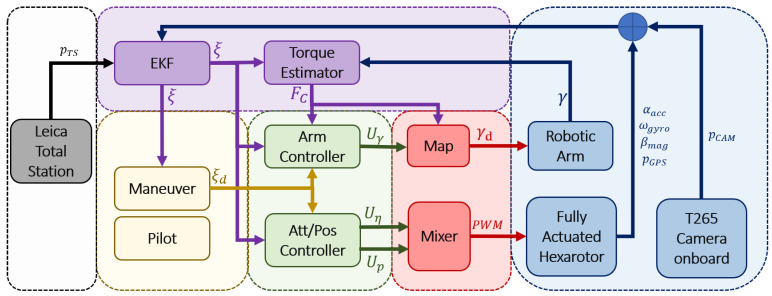
Control architecture.

The full control scheme is shown in Figure 14, and the control equations of the aerial platform are presented from (25) to (31):

(25) $$U_{p}=m\left[ge_{3}+K_{p}e_{p}+K_{v}e_{v}+L_{p}X_{p}+{\ddot{p}}_{d}\right]+G_{p}+C_{p}+{\tilde{N}}_{p\ddot{\gamma }}-F_{C_{p}}$$

(26) $$U_{\eta }=I_{\eta }\left[K_{\eta }e_{\eta }+K_{\dot{\eta }}e_{\dot{\eta }}+L_{\eta }\chi_{\eta }+{\ddot{\eta }}_{d}\right]+C_{\eta }+G_{\eta }+{\tilde{N}}_{\eta \ddot{\gamma }}-F_{C_{\eta }}$$

(27) $$U_{\gamma }=J_{E}^{T}M_{M}e_{\gamma }+C_{M}+G_{M}-J^{T}F_{d}+M_{QM}^{T}\left[\begin{array}{c} \ddot{p}\\ \ddot{\eta } \end{array}\right]-F_{c_{\gamma }}$$

(28) $$e_{p}=p-p_{d};\;e_{v}=k_{p}e_{p}+\lambda_{p}\chi_{p}+v_{d}-v$$

(29) $$e_{\eta }=\eta_{d}-\eta ;\;e_{\dot{\eta }}=k_{\eta }e_{\eta }+\lambda_{\eta }\chi_{\eta }+{\dot{\eta }}_{d}-\dot{\eta }$$

(30) $$e_{\gamma }=I\ddot{X}+k_{p}\left(X_{d}-X\right)+k_{v}\left({\dot{X}}_{d}-\dot{X}\right)$$

(31) $$F_{d}=F_{s_{d}}+k_{f}\left(F_{s_{d}}-F_{s}\right)$$

$U_{p}$, $U_{\eta }$, and $U_{\gamma }$ are the position, attitude, and arm joints control inputs, the values of $K_{i}$ are defined such that $K_{n}=1-\lambda_{n}^{2}+k_{n}$, $K_{\dot{n}}=k_{n}+k_{\dot{n}}$ and $L_{n}=-k_{n}\lambda_{n}$, are positive constant controller gains, $e_{p}$, $e_{v}$, $e_{\eta }$, $e_{\dot{\eta }}$, and $e_{\gamma }$ are the tracking errors, $\chi_{i}$ are the integral tracking errors, $J_{E}^{T}$ is the Jacobian of the manipulator, *X* represents the position of the end effector in the Earth fixed frame $\left\{E\right\}$ and $p_{d}$, $v_{d}$, $\eta_{d}$, $X_{d}$, and $F_{s_{d}}$ are the commanded references. ${\tilde{N}}_{p\ddot{\gamma }}$ and ${\tilde{N}}_{\eta \ddot{\gamma }}$ introduce the estimation of the dynamic coupling, which is considered negligible due to the low inertial and lightweight manipulator design. $I_{\eta }$ represents the submatrix of M(ξ) that govern the attitude dynamic. The rest of the terms $C_{p}$, $C_{\eta }$, $C_{M}$, $G_{p}$, $G_{\eta }$, and $G_{M}$ represent the terms of the Coriolis and the gravity matrix that affects each variable.

For a better understanding of how the control law is obtained and how it achieves the origin being Globally Uniformly Asymptotically Stable (GUAS), the derivation of the roll tracking controller $\phi_{d}$ is briefly explained below.

Defining $z_{1}=\phi_{d}-\phi$ as a tracking roll error, a candidate Lyapunov function is:

(32) $$V_{1}=\frac{1}{2}z_{1}^{2}+\frac{1}{2}\lambda_{1}\chi_{1}^{2}$$

where $\chi_{1}=\int_{0}^{t}z_{1}\left(\tau \right)\;d\tau$ is the integral error. Now, we choose $\dot{\phi }$ as a virtual control, and find its value to stabilize $V_{1}$. Derivating (32) and making $\dot{V_{1}}$ non-positive, we have:

(33) $$\dot{V_{1}}=z_{1}\left(\dot{\phi_{d}}-\dot{\phi }+\lambda_{1}\chi_{1}\right)$$

Therefore, $\dot{\phi }$ would be $\dot{\phi }=\dot{\phi_{d}}+\lambda_{1}\chi_{1}+k_{1}z_{1}$, the derivate of Lyapunov function $\dot{V_{1}}$ would be non-positive, and $V_{1}$ would be bounded. Furthermore, applying LaSalle–Yoshizawa Lemma, it proves the regulation of $z_{1}$:

(34) $$\dot{V_{1}}=-k_{1}z_{1}^{2}\leq W\left(z_{1}\right)$$

(35) $$\lim_{t\to +\infty }W(z_{1})=0$$

Equation (35) implies that $z_{1}$ would tend to zero. However, $\dot{\phi }$ is only a state and not a control input. Therefore, we define the stabilizing function α and the error between this function and the virtual control as:

(36) $$\alpha =\dot{\phi_{d}}+\lambda_{1}\chi_{1}+k_{1}z_{1}$$

(37) $$z_{2}=\alpha -\dot{\phi }$$

Now, we construct a control Lyapunov function for the entire system, where the error between the stabilizing function and the virtual control be penalized. Therefore,

(38) $$V_{2}=\frac{1}{2}z_{1}^{2}+\frac{1}{2}\lambda_{1}\chi_{1}^{2}+\frac{1}{2}z_{2}^{2}$$

Derivating (38), we have:

(39) $$\dot{V_{2}}=-k_{1}z_{1}^{2}+z_{1}z_{2}+z_{2}\left(\dot{\alpha }-\ddot{\phi }\right)$$

In (39), it can be seen that, if $\dot{\alpha }-\ddot{\phi }=-k_{2}z_{2}-z_{1}$, the derivate of the Lyapunov function $\dot{V_{2}}$ would be non-positive and, therefore, $V_{2}$ is bounded. In addition, applying LaSalle-Yoshizawa Lemma, the regulation is also proved:

(40) $$\dot{V_{2}}=-k_{1}z_{1}^{2}-k_{2}z_{2}^{2}\leq W\left(z_{1},z_{2}\right)$$

(41) $$\lim_{t\to +\infty }W(z_{1},z_{2})=0$$

To comply $\dot{\alpha }-\ddot{\phi }=-k_{2}z_{2}-z_{1}$, after some mathematical operations, the following expression can be obtained:

(42) $$\ddot{\phi }=\ddot{\phi_{d}}+\left(1-k_{1}^{2}+\lambda_{1}\right)z_{1}+\left(k_{1}+k_{2}\right)z_{2}-k_{1}\lambda_{1}\chi_{1}$$

Proceeding in the same way with the pitch and yaw, similar expressions can be obtained:

(43) $$\ddot{\theta }=\ddot{\theta_{d}}+\left(1-k_{3}^{2}+\lambda_{2}\right)z_{3}+\left(k_{3}+k_{4}\right)z_{4}-k_{3}\lambda_{2}\chi_{2}$$

(44) $$\ddot{\psi }=\ddot{\psi_{d}}+\left(1-k_{5}^{2}+\lambda_{3}\right)z_{5}+\left(k_{5}+k_{6}\right)z_{6}-k_{5}\lambda_{3}\chi_{3}$$

By introducing the expressions (42), (43) and (44) in the dynamic Equation (18), the expression of the control law (26) is deduced.

## 5. Experimental Results

This section aims to validate the performance of the fully-actuated platform and its capability to carry out the inspection operations in an outdoors scenario. First, the experiment consisted of analyzing the model estimated to be used in the controller. Second, a test bench experiment shows the capabilities of the robotic arm in a controlled environment that simulate the use case. Third, the advantages of using a platform with tilted rotors are shown comparing the results obtained of this fully-actuated system with a similar platform that implements the standard cascade linear controller. Finally, the full system is deployed and validated carrying out an inspection by contact operation in a bridge outdoors.

**Figure 15:**
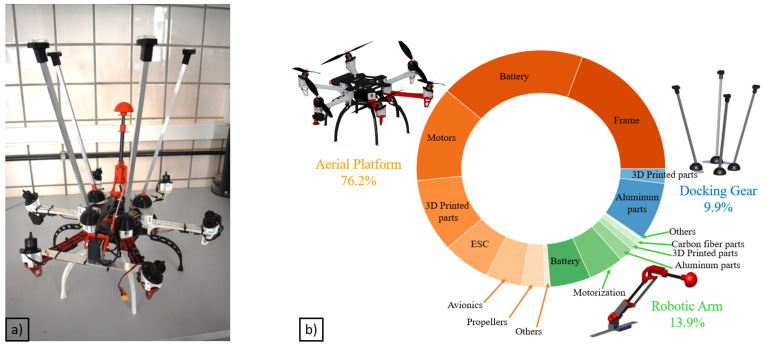
Fully actuated platform: (**a**) system integration; (**b**) mass distribution of the system.

The prototype used along these experiments is presented Figure 15, along with the final mass specifications.

### 5.1. Model Validation

Due to the controller implemented being based on a model estimation, the first step consisted of carrying out a model identification. To achieve this goal, all flight data such as control inputs, angular velocities, etc, were logged implementing a basic linear controller. During the flight, it was necessary to make aggressive maneuvers to excite the dynamics in such a way that the order of magnitude of any variable in the system is much greater than the standard deviation of the intrinsic noise to it.

**Figure 16:**
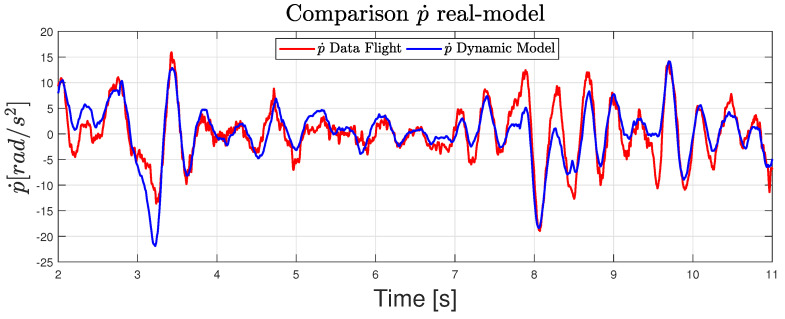
Comparison of experimental and dynamic model on roll attitude.

After logging the data, a filtered version of the signals and the different derivatives that appear in the model were obtained, such as angular acceleration from the gyro data. Then, applying the equation of the dynamic model (18), the angular acceleration can be obtained for each sample time. Figure 16 shows that the real and estimated angular pitch acceleration match fairly well.

Repeating this procedure with several configuration of the arm angles $\gamma_{1}$ and $\gamma_{2}$, the results were as good as the ones shown in the previous figure. Therefore, the dynamic model and the parameters estimation were considered validated.

### 5.2. Inspection in the Test Bench

**Figure 17:**
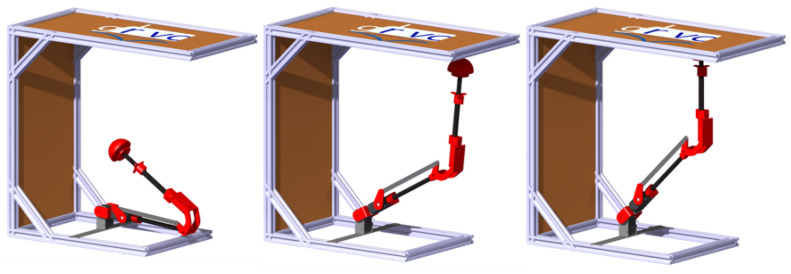
Test bench for the robotic arm.

This section aims to demonstrate the inspection capability of the robotic arm, before its installation on the hexarotor. For this purpose, an inspection has been done on a test bench on which the arm has been installed (Figure 17).

The inspection process of a vertical surface consists of different phases. First, the robotic arm is in the rest position ($\gamma_{1}=95^{\circ }$ and $\gamma_{2}=152^{\circ }$). The robotic arm then moves to the inspection position ($\gamma_{1}=61^{\circ }$ and $\gamma_{2}=55^{\circ }$). In this position, the robotic arm is close to the surface but does not touch it. Finally, the inspection takes place, during which the robotic arm performs a force control.

**Figure 18:**
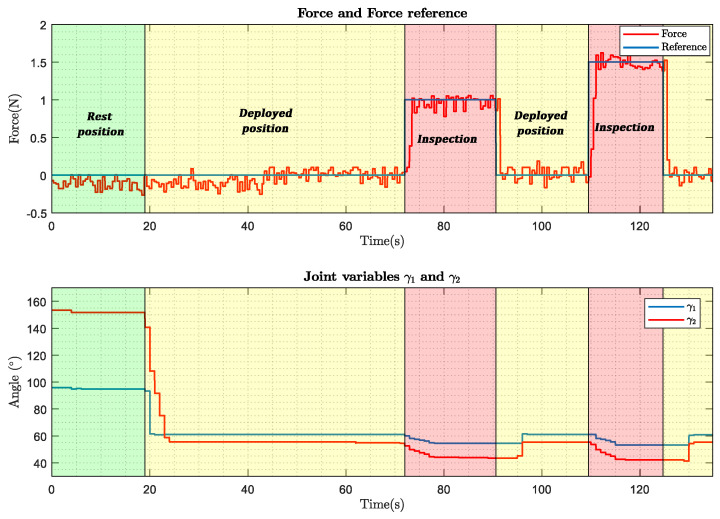
Inspection in the test bench.

Finally, Figure 18 shows the values of the articular variables, as well as the force demanded during an inspection of a vertical surface carried out on the test bench. This test simulates the force required to stick a sensor using a two-component glue.

### 5.3. Tilted Rotor Capabilities

In order to compare the differences between a fully-actuated platform and an under-actuated robot, three different flight modes have been implemented in the controller. In the first mode, called “standard stabilization”, the multirotor has a standard cascade linear controller without taking into account the inclination of the propeller, and then without the capability of accomplishing horizontal and angular movements independently. In contrast, the “command position” and “command angle” mode has been implemented to be able to command specific positions or angles in an independent way.

**Figure 19:**
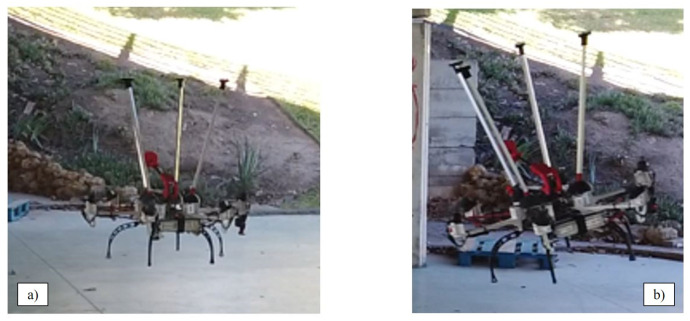
Fully actuated platform maneuvers. (**a**) during a planar translation (“com. position” flight mode); (**b**) tilted without losing the position reference (“com. angle” flight mode).

**Figure 20:**
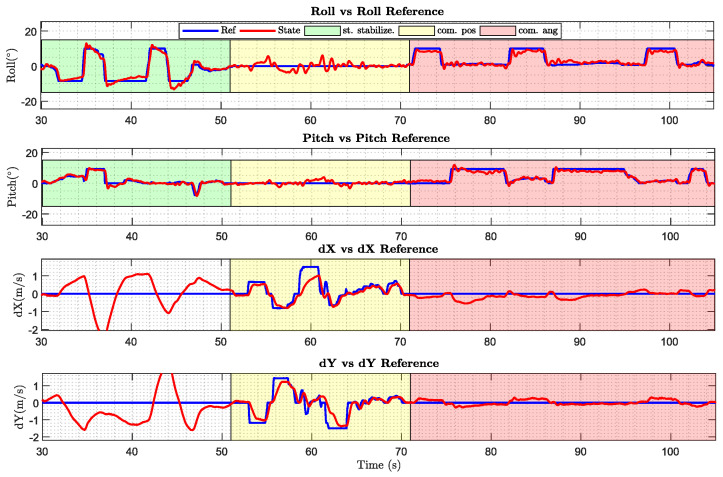
Performance of the fully-actuated platform in each flight mode implemented.

Figure 19 collects some pictures of the maneuvers that the fully-actuated platform is able to perform. Moreover, telemetry results of these maneuvers are also shown in Figure 20. The green background is the aforementioned "standard stabilization" mode in which is not possible to decouple the angle and position dynamics, since there is not a position controller implemented in this mode, so the dX and dY results do not have to match the reference. Yellow and red backgrounds show the fully-actuated modes in which the hexarotor is able to perform maneuvers in position and angles in an independent way. In yellow, the controller is focused on controlling the position and velocity without changing the attitude (“command position”), and in red the aerial platform controls the attitude maintaining a constant position. These results show how the platform is able to tilt 10 degrees without changing the position thanks to the full-actuation capabilities.

### 5.4. Inspection Experiments

**Figure 21:**
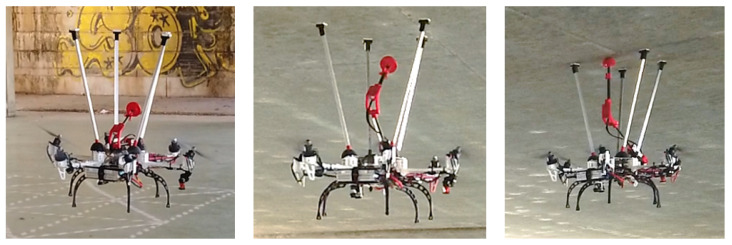
Sequence of a bridge inspection operation with the system proposed.

**Figure 22:**
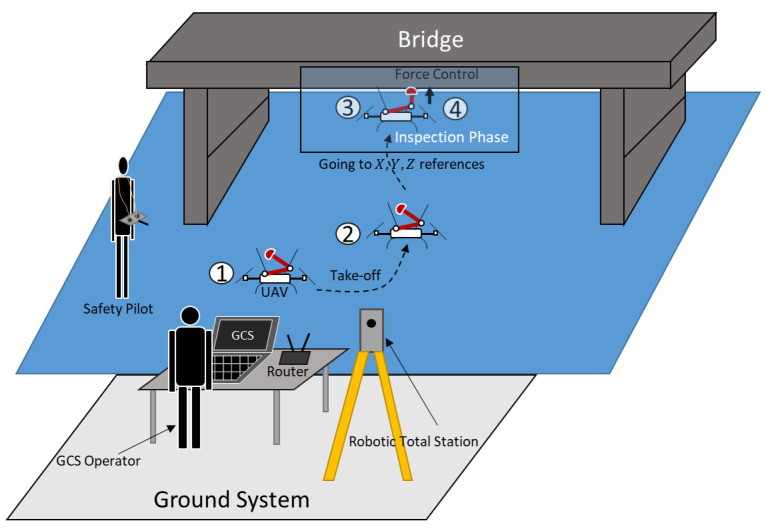
Scheme of the typical experiment.

This section aims to demonstrate the applicability that these kinds of platforms have in the field of bridge inspection by contact. The experiment carried out is illustrated in the sequence and scheme of Figure 21 and Figure 22. The experiment is carried out in “command position” flight mode presented in the previous section. The aircraft is commanded to follow references in *X*, *Y*, and *Z* coordinates until it reaches the bridge surface. Once the docking gear is in contact with the bridge surface, the phase called “inspection phase” starts. During this phase, the robotic arm is commanded to exert a force on the surface, for example, to install a vibration sensor, take measurements of the bridge, or accomplish a maintenance operation. Finally, the arm is commanded to stop the operation and the platform lands on a safe place.

**Figure 23:**
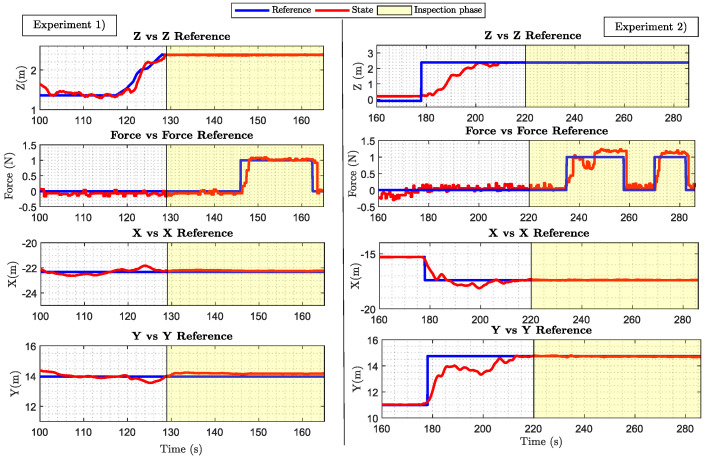
Experimental results.

Figure 23 illustrates the performance of the aerial manipulator in two different experiments. It shows the results in terms of how the controllers follow the values commanded in *X*, *Y*, *Z*, and $F_{C}$ during two different experiments. The area colored yellow in each result represents the inspection phase which starts when the docking gear is in contact with the bridge. It clearly shows that the system proposed is very stable during the inspection operation; moreover, they validate the feasibility of applying the robotic system proposed to the field of the bridge inspection operation using UAVs. A video compilation with one of the experiments can be found in [58].

## 6. Conclusions

This paper has presented a fully actuated aerial platform with a lightweight robotic arm for contact inspection of bridges and tunnels. The use of a multi-directional thrust aerial platform shows advantages over standard co-planar multirotor configurations for flying in the confined spaces under bridge decks and inside tunnels. Another advantage is the capability of applying forces in the horizontal plane of the presented prototype using its arm and the force sensor at the end effector, and without needing to tilt the platform for exerting lateral forces.

The presented prototype includes a new concept of docking gear which also provides an advantage during the inspection, getting the best accuracy during the contact operation, while facilitating the control strategy due to the decoupling between the flight and the inspection, and increasing safety during the operation. These aspects have been considered by the end-users as mandatory and are essential for proper commercial exploitation in bridge maintenance programs.

The localization system proposed is able to operate in GNSS-denied conditions and solves common problems that arise in several inspection applications. In those, it is essential to be able to visit the same points in different inspection operation to assess and trace how some specific defects evolve along the time.

The prototype as well as the localization system and the control technique implemented have been evaluated in a real bridge inspection which is a GNSS-denied environment and uses a robotic total station and a reflector prism on-board as position estimation to close the position control loop and inspection sensor.

This work demonstrates that it is possible to solve the time-consistent reference frame issue that affects most of the inspection applications using UAVs in a GPS denied environment. This has been considered as one of the most important requirements imposed by the need for following the evolution of the defects over time. It also shows the advantages of using a fully-actuated platform against a conventional co-planar multirotor to accomplish an inspection by contact application one more time. It is clear that those platforms have enhanced capabilities in applications that require exerting forces with a flying robot. Finally, it has been shown that using a docking gear can improve the measurements obtained thanks to the stability provided by maintaining a contact with the infrastructure.

As future work, the prototype will be enhanced to inspect pillars and tunnel walls, including an adjustable docking gear, and the multi-sensor localization system will increase the number of sensors and its technology.

## Figures and Tables

**Table 1:** Rotation angles.

i	1	2	3	4	5	6
$\alpha_{i}$	$-20^{\circ }$	$20^{\circ }$	$-20^{\circ }$	$20^{\circ }$	$-20^{\circ }$	$20^{\circ }$
$\beta_{i}$	$20^{\circ }$	$-20^{\circ }$	$20^{\circ }$	$-20^{\circ }$	$20^{\circ }$	$-20^{\circ }$

**Table 2:** Main features of the fully-actuated platform.

Weight	2.00 kg
Payload	∼ 600 g
Maximum achievable lateral forces	$F_{x}$ = 415 g $F_{y}$ = 555 g
Propellers	DJI 9 × 4.5 in
Battery	4S 5300 mAh
Rotation angles	$\alpha =20^{\circ }$ $\beta =20^{\circ }$

**Table 3:** Parameters of the linear regression.

$m_{1}=-0.0031661$	$n_{1}=16.368$
$m_{2}=-0.0088239$	$n_{2}=31.674$
$ADC_{limit}=2705$	

**Table 4:** Main features of the robotic arm.

Weight	0.370 kg
Dimensions	Link 1 = 25 cm Link 2 = 20 cm
Rotation range	Shoulder pitch = [−100${}^{\circ }$, 100${}^{\circ }$] Elbow pitch = [20${}^{\circ }$, 160${}^{\circ }$]
Battery	2S 1300 mAh
